# Improving rice population productivity by reducing nitrogen rate and increasing plant density

**DOI:** 10.1371/journal.pone.0182310

**Published:** 2017-08-02

**Authors:** Guangli Tian, Limin Gao, Yali Kong, Xiangyu Hu, Kailiu Xie, Ruiqing Zhang, Ning Ling, Qirong Shen, Shiwei Guo

**Affiliations:** Jiangsu Provincial Key Lab for Organic Waste Utilization, National Engineering Research Center for Organic-based Fertilizers, Jiangsu Collaborative Innovation Center for Solid Organic Waste Resource Utilization, Nanjing Agricultural University, Nanjing, China; Wageningen University, NETHERLANDS

## Abstract

In terms of tillering potential, the aboveground portions of rice are significantly influenced by the nitrogen level (NL) and transplant density (TD). To obtain a suitable combination of NL and TD, five NLs (0, 90, 180, 270 and 360 kg ha^-1^) and two TDs [high density (HD), 32.5×10^4^ hills ha^-1^; low density (LD), 25.5×10^4^ hills ha^-1^] were used in the rice experiments during 2012 to 2014, in Jiangsu, China. The results showed the highest grain yield of rice obtained at HD and LD when N supply was 180 and 270 kg ha^-1^, respectively. That’s because there are more tillers per unit area, a larger leaf biomass fraction of total aboveground biomass, a larger leaf area index (LAI) and a larger canopy photosynthesis potential (CPP) at HD. It can be concluded that, higher rice planting densities resulted in less N inputs, while more N is needed to improve single plant actual tiller ability under low density to offset the reduced planting density. When the NL was more than 180 kg ha^-1^, the actual tillering ability of a single plant at LD was 20% more than that at HD. Based on these results, the supply of 1 kg N can be replaced by adding approximately 1000 planting hills per hectare. Therefore, adjusting the transplant density could be an efficient method to reduce the amount of nitrogen fertilizer and increase the nitrogen fertilizer use efficiency, which is very conducive to the sustainable development of agriculture.

## Introduction

Rice (Oryza sativa L.) is one of the most important food crops and is the staple food for more than half of the world population [1]. Due to increasing global population and improvements in the quality of life, approximately 50% more food will be needed by 2030, with double that being needed by 2050 [2]. Enhancement of N use efficiency is a key factor to sustain and increase the yield of rice. Due to its main role in the formation of chlorophyll, proteins and nucleic acids, N is considered a major mineral element that is required more consistently and in larger amounts than the other nutrients for rice production [3, 4]. To obtain ideal population productivity, nitrogen fertilizer has been widely used by farmers, while the adjustment of planting density is often neglected. In China, large amounts of N (approximately 420 kg N ha^-1^yr^-1^) have been applied, especially for rice-wheat rotation, with approximately 550 kg N ha^-1^yr^-1^ in the Jiangsu province alone [5, 6]. However, excess nitrogen applications may not improve the population productivity accordingly, resulting in reduced nitrogen use efficiency and even in acidification of the soil [7, 8]. Reasons for explaining why the rice population productivity does not increase under such high nitrogen supplies have been widely investigated. However, most studies have shown that later maturity, plant lodging, lower filling percentages and ineffective tillers often occur under high nitrogen supply due to the extended vegetative growth relative to reproductive growth [9–11]. According to the regularity of nitrogen requirements and biomass accumulation in rice, previous studies have shown that the optimal management of N applications at different growth stages might regulate the source–sink relationship, which then can further increase rice yields [12]. However, due to the difficulties in accurately predicting N fertilizer requirements, N supply rates exceeding plant requirements are often applied, especially in China, where overuse of N fertilizer is widespread [6, 13].

Generally, another approach to obtain ideal population productivity is to increase the TD. Rice is a unique perennial crop with an indeterminate tillering potential, and the actual tillering number is easily influenced by nutrients availability, planting density and variety [14–16]. Both nitrogen accumulation and tillering capacity were significantly influenced when the TD was varied from 5 seeds square^-1^ to 125 seeds square^-1^ [17]. Higher densities might also increase pre-silking N uptake with no significant effects on post-silking N uptake due to the overall possession of larger aboveground biomass [18]. Previous studies have shown that having more aboveground biomass could compensate for the relative shortage in nitrogen supply needed to obtain ideal production [15]. However, sufficient leaf area (LAI) is a necessary condition for biomass production, which are seriously affected by the NL and TD. The effective rice LAI has been shown to change among regions by as much as 6 to 10, and the related parameters, such as the extinction coefficient and intercepted photosynthetically active radiation, changed along with that of the LAI [19]. The population productivity increased at optimum planting densities. Beyond the optimum density, the population productivity remained constant, or even decreases, as LAI continues to increase.

The interaction between the NL and TD is different among diverse plant groups. For smaller groups, N improves the rice population productivity mainly by increasing tillers number. When the rice group size is optimal, the improved population productivity mainly depends on optimizing the combination of maximum tillers and numbers of spikelets per panicle. When the aboveground biomass is too large, improving the rice grain yield mainly depends on increasing the number of spikelets per panicle [17]. Generally, there is a negative relationship between the number of panicles per unit area and the number of spikelets per panicle [12]. However, whether the proposed N management regimes can be used for different planting densities remains uncertain. Consequently, the appropriate combination of TD and corresponding NL could increase the population productivity, and rice grain yield might improve significantly.

The goals of this study were to investigate the characteristics of population productivity under different N supplies and transplant densities, and to provide a theoretical basis for choosing a suitable combination of NL and TD, ultimately to simultaneously improve rice grain yield and N efficiency by regulating the NL and TD. As such, field experiments were conducted from 2012 to 2014 in attempt to elucidate the interrelationship between NL and TD.

## Material and methods

### Experimental design

Field experiments were conducted in 2012, 2013 and 2014 at the Institute of Agriculture Science Research at Rugao Counties (32°44′N, 120°49′E), Jiangsu Province, China. The soil type was a high sandy loam with the following basic physical and chemical properties at a 0–20 cm depth: (1) 14.49 g kg^−1^ of organic matter, (2) 1.52 g kg^−1^ of total N, (3) 8.40mg kg^−1^ of available phosphate, (4) 78.40 mg kg^−1^ of available potassium, (5) and pH 7.50.

In the field experiments, five N levels were applied as the main treatments, and two planting densities were designated subtreatments. The size of the plot was 50 m^2^. The treatments were arranged in a randomized block design with three replicates. The planting densities of 32.5 × 10^4^ (HD) and 25.5 × 10^4^ (LD: based on the practices of local farmers) hills ha^-1^ were used with two seedlings per hill. The total N supply amounts of the five N rates were 0, 90, 180, 270 and 360 kg ha^-1^, which were applied according to the 2.5:2.5:3:2 ratio at the basal, tillering, panicle initiation and spikelet differentiation stages, respectively. In all treatments, phosphorus (75 kg of P_2_O_5_ ha^-1^) was the base fertilizer, while potassium (90 kg of K_2_O ha^−1^) was applied at the transplant (60 kg ha^-1^) and panicle initiation stages (30 kg ha^-1^). Field management and irrigation followed local high-yielding practices. Insects were intensively controlled using chemicals to avoid biomass and yield losses.

The tested cultivar *Zhendao 11* is an inbred and early maturing late *japonica* rice cultivar in Jiangsu province that is a popular variety among local farmers. Each year during the study, pre-germinated seeds were sown on a seedbed on about May 15^th^, and then seedlings with 4 leaves were transplanted on about June 15^th^. When transplanted, a labeled rope was used to make transplanting space according to the following experimental design: the row pitch × spacing in the rows was 22 × 13 cm and 28 × 13 cm for the HD and LD seedings, respectively.

### Sampling and measurements

In each plot, ten hills were marked to count the tillers (including the main stem) starting at 10 days after transplantation at 7-day intervals until the flowering stage. A tiller with at least one visible leaf was counted.

Samples were collected from the ground level at the following five growth stages: mid-tillering, max-tillering, booting, flowering and harvest. We sampled three hills per plot during each sampling event, and each treatment contained three replications. After cleaning, the aboveground samples were divided into leaves, sheaths, stems and panicles. Each part was oven-dried at 105°C for 30 min and then at 70°C to achieve a constant weight, after which they were digested with H_2_SO_4_–H_2_O_2_ at 260–270°C. An Auto Analyzer 3 Digital Colorimeter (Bran + Luebbe Inc., Norderstedt, Germany) was used to determine the total N concentration.

### Leaf area index (LAI) measurement

The LAI was calculated by dividing the measured leaf area by the ground surface area. We computed the area of each leaf from one tiller based on the length-width method with the following formula: Leaf area (cm^2^tiller^-1^) = K × L × W, where K is the adjustment factor with the value of 0.75, L is the total leaf length and W is the total leaf width from one tiller. The LAI was then calculated as follows: LAI (m^2^ m^−2^) = [A (cm^2^) × tillers (m^−2^)] */* 10000, where A is leaf area multiplied by number of tillers, in m^-2^.

### Net photosynthesis rate measurement

At every growth stage, net photosynthesis rate was measured on the newly expanded leaves on sunny days between 9:00 to 15:00 with a portable photosynthesis system (LI-6400XT; LI-CORInc., Lincoln, NE, USA). Though out all measurements, leaf-air vapor pressure deficit (VPD) was controlled at (1.77±0.37) kPa. In the leaf chamber, CO_2_ concentration was about (379±14) μmol mol^–1^, the leaf temperature was set to 30–35°C, the relative humidity was 45% and the photosynthetic photon flux density was 1500 μmol m^-2^ s^-1^, The data were recorded after equilibration to a steady state.

### Calculation methods

$$\text{Agronomic efficiency of N }\left({\text{AE}}_{\text{N}}\right){\text{ kg kg}}^{-1}=(\text{Nitrogen plot yield}-\text{No nitrogen plot yield})/\text{Nitrogen supply rate}.$$

$$\text{Yield contribution of N }\left({\text{YC}}_{\text{N}}\right)\%=\left(\text{Nitrogen plot yield}-\text{No nitrogen plot yield}\right)/\text{Nitrogen plot yield}\times 100.$$

$$\text{Canopy photosynthesis potential }\left(\text{CPP}\right){\text{ mol CO}}_{2}{\text{ ha}}^{-1}{\text{ s}}^{-1}=\text{Unit leaf photosynthetic rate}\times \text{leaf area}.$$

### Statistical analysis

The differences between the treatments were compared using the least significant difference test with a 5% level of probability using SPSS 16.0 software. Linear regression analysis and figures was performed by using SigmaPlot 12.5. Estimation of the minimum amount of nitrogen fertilizer was completed using a model from the linear plus platform in the SAS software.

## Results

### Grain yield and its mainly component parameters

The N supply rate had a significant effect on the grain yield during all three years (Table 1). The results showed that rice plants at high and low densities obtained their highest grain yields when they needed lower and higher nitrogen supply rates, respectively. At the same time, there were no significant differences in the highest grain yield between the two different densities. However, all grain yield components (the number of panicles per unit area, the number of spikelets per panicle, the filled grain rate and 1000-grain weight) were significantly affected by NL (Table 1). The largest average range in variation was for the number of panicles per unit area, which was approximately 80%, followed by the number of spikelets per panicle (approximately 16.4%), grain weight (approximately 15.9%) and filled grain rate (7.5%). In addition, the TD had an impact on the number of panicles per unit area and the number of spikelets per panicle, but not on the filled grain rate and grain weight.

**Table 1:** Grain yield, panicles, spikelets per panicle, filled grain rate and 1000-grain weight of rice under different N levels and transplant densities during 2012 to 2014.

Year	N level (kg ha^-1^)	Grain Yield (t ha^-1^)		Panicles Number (10^4^ ha^-1^)		Spikelets Number (panicle^-1^)		Filled Grains rate (%)		1000-Grain Weight (g)	
		HD	LD	HD	LD	HD	LD	HD	LD	HD	LD
2012	CK	6.0c	6.2c	194f	201f	124ab	108cd	98.2a	98.4a	29.8a	29.1a
	N90	9.0ab	8.7b	284d	251e	125ab	128ab	98.0a	97.5ab	26.7b	26.9b
	N180	9.2ab	9.2ab	320c	285d	112bc	131a	97.3ab	96.6b	25.8c	26.3bc
	N270	8.6b	9.6ab	336b	339b	125ab	119ab	91.0e	92.4d	25.5cd	24.8d
	N360	8.9ab	9.0ab	369a	329b	110cd	106d	94.0c	94.0c	25.5cd	24.7d
2013	CK	5.7e	5.7e	194f	192f	132b	136ab	95.2a	94.4ab	26.8a	26.6ab
	N90	7.5d	7.4d	266e	249e	140ab	147a	96.5b	93.7ab	26.6ab	26.5a
	N180	8.1bc	7.9c	291d	282d	146a	147a	94.7ab	96.0a	25.3b	25.3b
	N270	8.1bc	7.8c	318bc	300bc	139ab	143ab	91.8cd	90.2bc	25.0bc	24.9bc
	N360	8.3ab	8.4a	351a	335a	139ab	133b	88.3e	91.0d	23.3d	24.2c
2014	CK	6.0c	5.8c	183d	167d	139bc	130d	97.8ab	98.2a	26.7a	27.4a
	N90	8.7b	8.6b	245b	230c	135cd	154b	97.6ab	97.9ab	26.5ab	26.4ab
	N180	9.9a	9.4ab	302ab	296b	144bc	154b	97.8ab	97.3ab	26.2ab	26.6ab
	N270	9.9a	10.0a	344ab	309ab	146bc	178a	97.4ab	96.5ab	24.1c	25.0bc
	N360	9.7a	10.1a	326ab	323ab	149bc	148b	96.6ab	96.2b	22.5d	22.6d
Statistics											
N level (N)		***		***		***		***		***	
Density (D)		ns		ns		ns		ns		ns	
Year		***		**		***		***		***	
N*D		ns		ns		ns		ns		ns	
N*Y		***		ns		***		***		***	
D*Y		ns		ns		ns		ns		ns	
N*D*Y		ns		ns		ns		**		ns	

Note: Different small letters indicate significant differences at *P*< 0.05, and analysis of variance results for individual indicators for each year. ns indicates not significant, *** and ** indicate significant at 0.001 and 0.05 probability levels, respectively.

### Aboveground biomass

**Fig 1:**
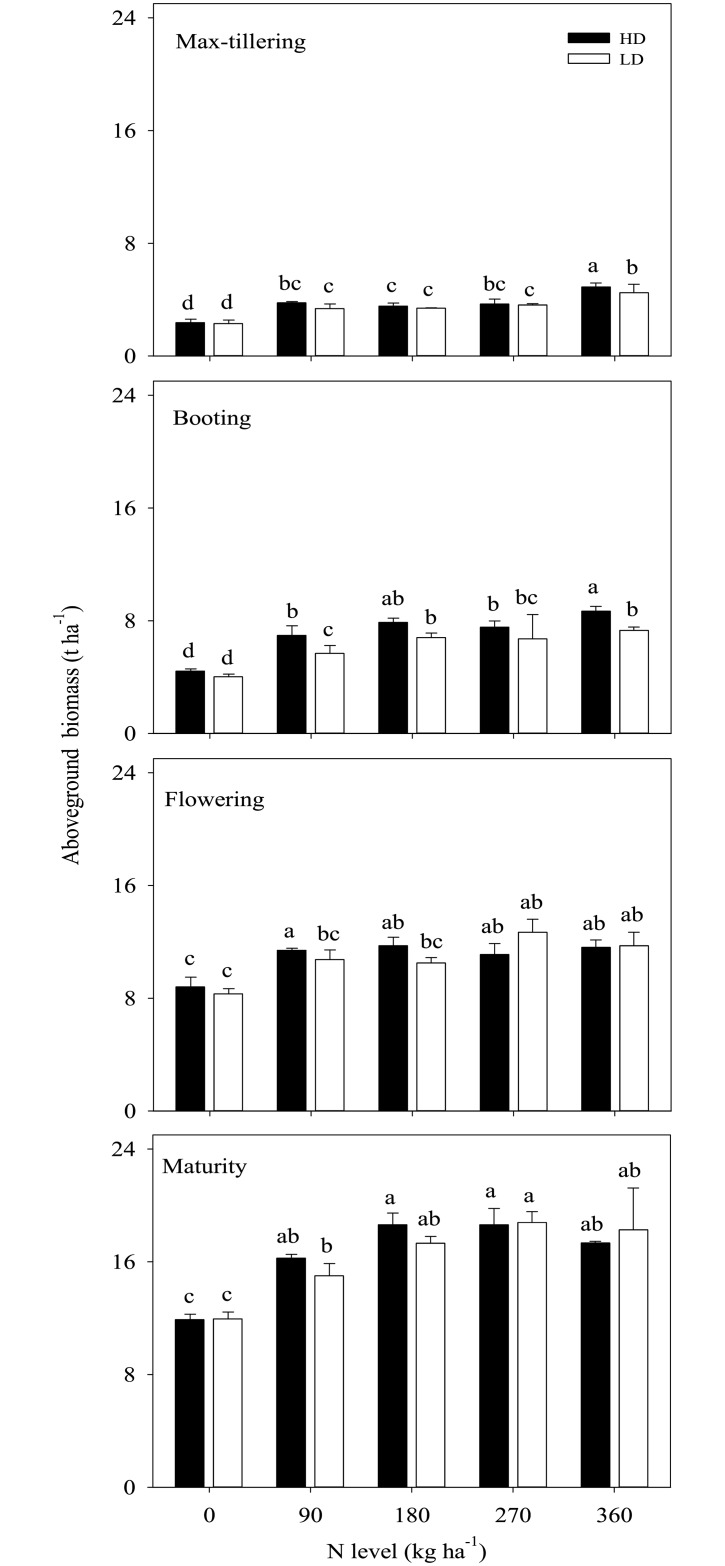
Effects of N level and transplant density on the aboveground biomass of rice at different growth stages during 2012–2014. The data are mean values ± SD. Different small letters in each group indicate significant differences at *P*< 0.05, and analysis of variance results at each growth stage.

At the different growth stages, the aboveground biomass showed different dynamic changes between the treatments (Fig 1). During the early stages, the aboveground biomass increased as the N supply amount increased, reaching 2.31–4.91 t ha^-1^ and 4.02–8.68 t ha^-1^ from 0 to 360 kg N ha^-1^ at max-tillering and booting stages, respectively, when little differences were observed between the two densities. Subsequently, the differences between densities disappear at the end of growth period. The highest biomasses were achieved with 180 and 270 kg ha^-1^ of N at the high and low densities, respectively. However, these results were confounded at the flowering stage, which might be mainly because the rice spikelets were filling earlier under N-deficient conditions than under N-rich conditions.

### Development features of the aboveground parts

**Fig 2:**
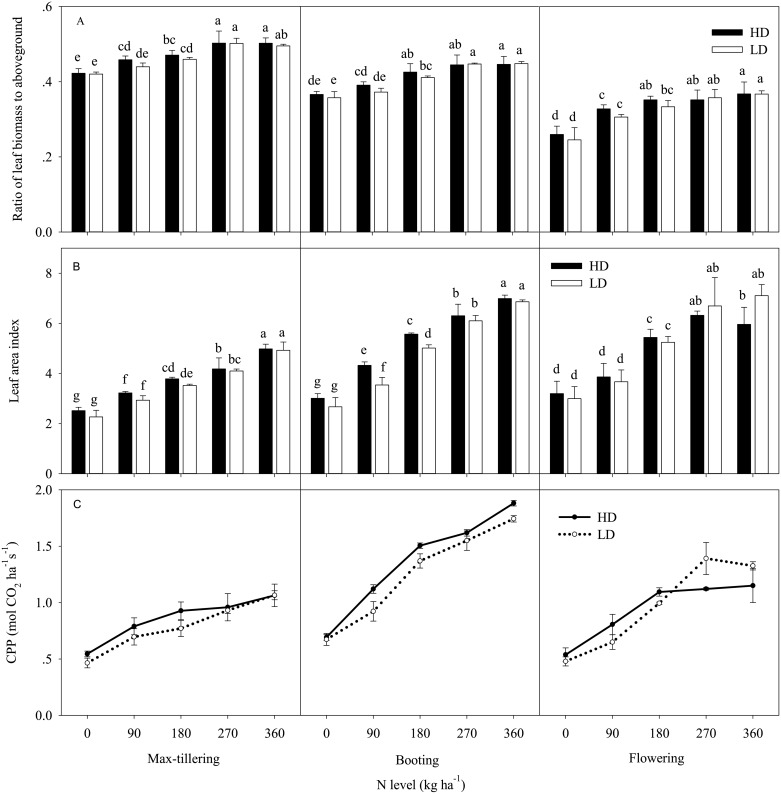
Effects of N level and transplant density on the leaf biomass fraction of aboveground (A), leaves area index (B), and canopy photosynthesis potential (CPP, C), at the max-tillering, booting, and flowering stages. The data are the mean value ± SD. Different small letters in each group indicate significant differences at *P*< 0.05.

Under the different treatments, the number of tillers and the development characteristics of the aboveground samples were significantly different (Table 1, Fig 2A). Further analysis the number of panicles per unit area under different treatments, indicated that the number of effective tillers increased with an increasing N supply rate. In addition, the tillering ability of rice seedlings from a single hill at LD was approximately 20% more than that at HD, which trend was similar to the ratio of basic hills at HD to that at LD. The ratio reached the largest value when the N supply rate was 270 kg ha^-1^. In contrast, the number of tillers per unit area, at HD the number was approximately 1.15-fold higher than at LD, and the ratio reached the largest value when the N supply rate was 180 kg ha^-1^.

The leaf biomass fraction of aboveground biomass increased with increasing N supply, and the ratio increased from 0.43 to 0.49 at the max-tillering stage, from 0.37 to 0.47 at booting stage and from 0.27 to 0.39 at flowering stage. The ratio decreased more under lower N supply than under higher N supply. In addition, when the N supplied was less than 180 kg ha^-1^, the ratio was higher at HD than at LD. Inversely, when the N supply rate was more than 180 kg ha^-1^, the leaf biomass fraction of aboveground biomass was not significantly different between the densities. Meanwhile, the effective leaf area index demonstrated the same trend among different treatments, though the difference became larger from the max-tillering to the flowering stage (Fig 2B). The canopy photosynthesis potential also showed a similar trend with the effective leaf area index. The largest differences between the two densities were observed at the flowering period (Fig 2C).

### N concentration, N accumulation and N efficiency

Firstly, the N concentrations in the different organs were clearly different. Even in the same organ, differences existed among the N treatments, but not between the densities (Table 2). The leaf N concentration was the highest among all organs, and its range was the largest. The leaf N concentration levels ranged from 22.9 to 29.3 mg g^-1^, 18.8 to 28.5 mg g^-1^, 15.8 to 30.0 mg g^-1^, and 5.3 to 9.4 mg g^-1^ during the stage of max-tillering, booting, flowering, and harvest, sequentially. The leaf N concentration declined during the entire growth period when the N supply rate was low, although leaf N concentration only declined at maturity under high N supply. In contrast, the N concentration ranges in the sheath were from 8.4 to 11.8 mg g^-1^, 5.8 to 10.2 mg g^-1^, 5.0 to 11.7 mg g^-1^, 3.6 to 9.8 mg g^-1^ and 2.6 to 6.3 mg g^-1^ at each stage, respectively, and the N concentration in the sheath decreased throughout the growth period. However, the largest variation in N concentration was during flowering stage at both densities. With respect to stem and panicle, the N concentration slightly decreased in the stem and slightly increased in the panicle at maturity.

**Table 2:** Effects of N level and transplant density on nitrogen concentration in the leaf, sheath, stem, and spike (mg g^-1^) of rice at different growth stages during 2012–2014.

N Level (kg ha^-1^)	Maximum-tiller		Booting		Flowering		Harvest	
	HD	LD	HD	LD	HD	LD	HD	LD
Leaf								
CK	22.9e	24.6de	19.7e	18.8e	15.8f	19.3e	5.3c	5.8c
N90	25.8cd	26.6bc	22.7d	22.8d	21.7d	20.1e	7.8b	7.6b
N180	25.9cd	28.5ab	24.1cd	22.4d	25.0c	28.0b	7.1b	8.1ab
N270	27.4ab	29.2ab	25.1bc	25.0bc	28.6b	27.9b	8.3ab	8.0b
N360	29.3a	27.0ab	26.5ab	28.5a	27.4b	30.0a	8.2ab	9.4a
Sheath								
CK	8.4f	8.6e	5.8e	5.9e	5.0e	5.5e	3.2de	3.0de
N90	10.1de	10.0cd	6.4e	6.7d	6.2d	5.8d	3.3d	2.6e
N180	9.5d	10.9bc	8.1bc	7.5c	7.6c	8.4c	4.6bc	4.1c
N270	10.9bc	11.1b	8.2bc	8.1bc	11.4a	10.3b	6.2a	4.8b
N360	11.8a	10.4cd	8.5b	10.2a	10.7a	11.7a	6.3a	5.1b
Stem								
CK	-	-	-	-	4.8f	5.5f	6.3cd	6.3cd
N90	-	-	-	-	7.3e	7.4de	6.9c	6.0de
N180	-	-	-	-	8.4de	9.1cd	5.4e	6.5cd
N270	-	-	-	-	10.8b	10.6bc	7.7b	6.9c
N360	-	-	-	-	12.0ab	13.2a	8.8a	8.0b
Panicle								
CK	-	-	-	-	9.1e	9.0e	11.0f	11.2f
N90	-	-	-	-	10.3d	10.0de	11.5f	11.9ef
N180	-	-	-	-	10.8d	11.0cd	12.6de	13.3cd
N270	-	-	-	-	11.9bc	12.7ab	13.6bc	14.0ab
N360	-	-	-	-	12.0ab	13.0a	14.8a	14.5ab

Note: Different small letters indicate significant differences at *P*< 0.05, and analysis of variance results for individual organs at each growth stage.

**Fig 3:**
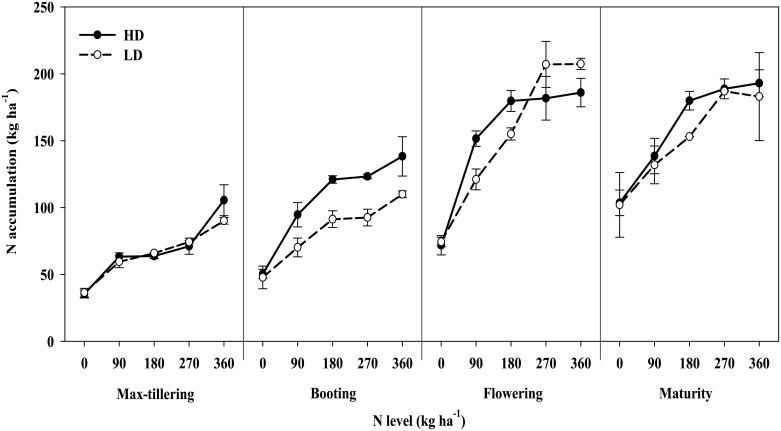
Effects of N level and transplant density on nitrogen accumulation in aboveground organs of rice at different growth stages. The data are the mean values ± SD.

Secondly, the N accumulation of aboveground parts was significantly (P < 0.05) affected by N supply at both densities, reaching the peak during the flowering stage (Fig 3). During the vegetative period, the N accumulation increased with increasing nitrogen supply. Moreover, the N accumulation was higher at HD than at LD, and the maximum difference occurred during the booting stage. The N accumulation did not increase or displayed a slight decrease in some of the individual treatments from the flowering stage to the harvest stage. The highest N accumulations at the high and low densities were 205 and 207 kg ha^-1^ under the 180 and 270 kg ha^-1^ N levels, which were obtained during the flowering and maturity stages, respectively, indicating that rice grown at a lower TD can absorb N longer.

**Fig 4:**
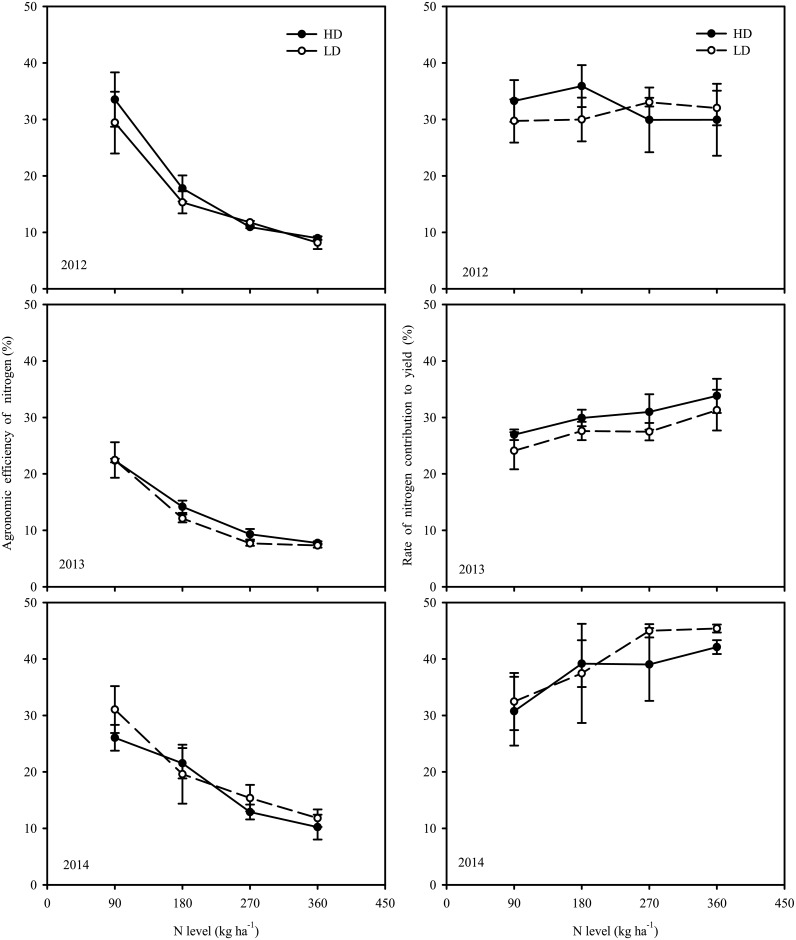
Effects of N level and transplant density on agronomic efficiency of N (AE_N,_ %) and yield contribution of N (YC_N_, %) of rice during 2012–2014.

Thirdly, the N use efficiency presented by AE_N_ (Fig 4). The AE_N_ was improved at lower nitrogen supply rate at HD than at LD; otherwise the AE_N_ was lower at HD than at LD under higher nitrogen supply rate. In addition, AE_N_ was significantly affected by the year. The rate of the nitrogen contribution to the yield (YC_N_) between the high and low planting densities showed a much larger difference (Fig 4). Though no significant difference was observed in the maximum value of YC_N_ between the high and low planting densities, the N supply needed to reach the maximum value was different (180 and 270 kg ha^-1^, respectively). At the same time, these results indicated that the N supply rate and experimental year had significant effects on the YC_N_, while the density had no significant effect on the YC_N_, *i*.*e*., there was no significant interaction among these three factors.

## Discussion

**Fig 5:**
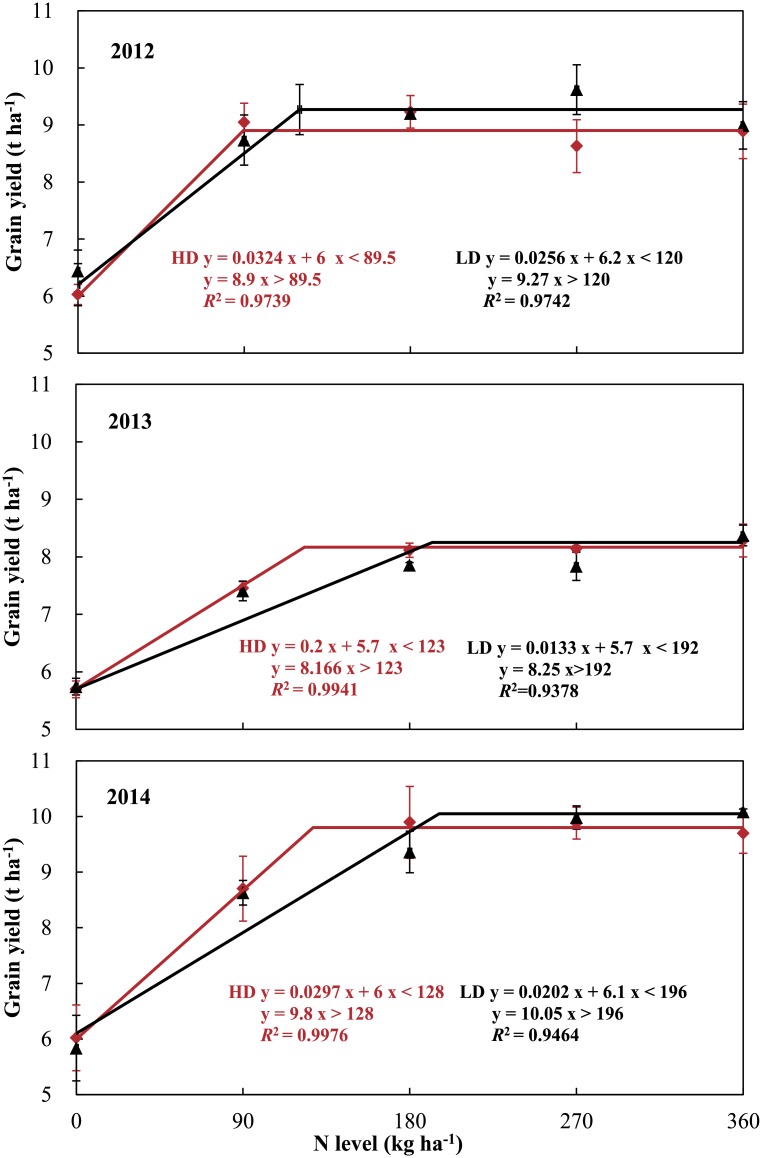
Estimation of the minimum amount of nitrogen fertilizer for the highest yield of rice at high density (red) and low density (black) separately using the model of linear plus platform in SAS software.

Consistent with previous studies, our results demonstrated that the response of rice yield to NL follows a parabolic curvilinear relationship both at HD and at LD [12, 20, 21]. The highest grain yields at HD and LD were achieved at lower and higher NLs, respectively. It is worth noting that lower rice grain yield was attributed to continuous high temperature during the flowering and grain-filling stages in 2013. Since high temperature impairs dry matter production and decreases rice grain size, the grain-filling rate and setting rate are negatively affected, ultimately resulting in decreased grain yield [22, 23]. According to our results, the minimum amount of nitrogen fertilizer for the highest yield under different TD was estimated by a linear plus platform model (Fig 5). The estimation results implied that 1 kg N ha^-1^ was saved when the number of hills increased by 2295, 1014 and 1029 hills ha^-1^ in 2012, 2013 and 2014, respectively. According to these data, the NUE at HD would be significantly higher than that at LD under lower N levels, especially when the NL was lower than 130 kg ha^-1^. However, the results showed no significant difference in the NUE between the HD and LD, while previous studies showed that plant density affected grain yields and nitrogen efficiency significantly in rice and other cereal crops [24, 25]. Therefore, this phenomenon might be attributed larger gradient interval of NL and smaller gradient interval of TD, which resulted in the mutual compensation between single and group development, *i*.*e*., mutual compensation between basic number of seedlings and number of tillers per unit area.

There are certain features that determine the actual tillering ability of rice, which are easily influenced by N levels and growing space [14, 16]. Our results also showed that the numbers of tillers were significantly affected by NL and TD before field spacing was closed. Moreover, the difference in tiller numbers between the densities decreased with the development of the panicle, which can be attributed to the higher percentage of earing tillers at LD than at HD, mainly due to larger living space, more intense radiation, time of tiller occurrence, higher N levels and other less-known factors [14, 26, 27]. In our results, there was no significant difference in the nitrogen concentration in each organ of the rice plants between the two densities during each growth stage (Table 2), demonstrating that the differences in actual tillering ability for a single hill between the two densities is mainly affected by living space, namely, planting density and light quality.

Considering the factors that influence population productivity, the leaf area index (LAI) plays an important role in radiation interception and carbon assimilation. LAI is regulated by the NL and the number of tillers per unit area. Thus, the interaction between the leaf nitrogen concentration and the number of tillers could be such that: when the plant population becomes too high, tiller death cannot be terminated by a very high leaf N concentration [28, 29]. As far as we know, the last two leaves of rice would grow, and the leaf area index of the rice population at the flowering stage would be larger than that at the booting stage. However, in our results, there were no significant differences between these two stages (Fig 2B), mainly due to the tiller death that offset the leaf area of the two last new leaves. At the same time, the corresponding leaf area index values for the highest grain yield at the two densities were not the largest, being approximately 6 and 7 at HD and LD, respectively. In addition, the need for N supply when the maximum effective leaf area reached was greater at LD. That is, the negative effect of LD on the tiller population or panicles per unit area should be offset by a higher N supply. Thus, a greater LAI is not always better because the relationship between LAI and radiation was not linear. Previous studies showed that a suitable LAI was suggested to be 5–7 during the flowering stage [28, 29]. Some studies have indicated that when the LAI is approximately 3, the radiation interception of PAR approaches 90%; any further increase in LAI would be null [30]. This effect on the LAI is because excessive LAI will attenuate the light intensity and/or because light quality changes at the base of the canopy, where tiller buds and young tillers are located [31].

It is known that CO_2_ assimilation mainly depends on green leaves, which produce more than 90% of crop biomass [21]. Thus, the optimal leaf biomass fraction of aboveground biomass might be an important candidate for determining yield. Our results showed that the leaf biomass fraction of aboveground biomass increased with N supply in a certain range and that the ratio is slightly higher at HD (Fig 2A). A suitable LAI (5–7) was obtained under appropriate N supply conditions (180 kg N ha^-1^) at HD, demonstrating that the rice canopy possesses the greatest canopy photosynthesis potential (CPP) under optimal combination of NL and TD (Fig 2C). At LD, the greatest CPP occurred at a higher NL (270 kg N ha^-1^), indicating that the capacity of biomass formation is determined by the interaction between LAI and LNC (Fig 2B, Table 2).

**Fig 6:**
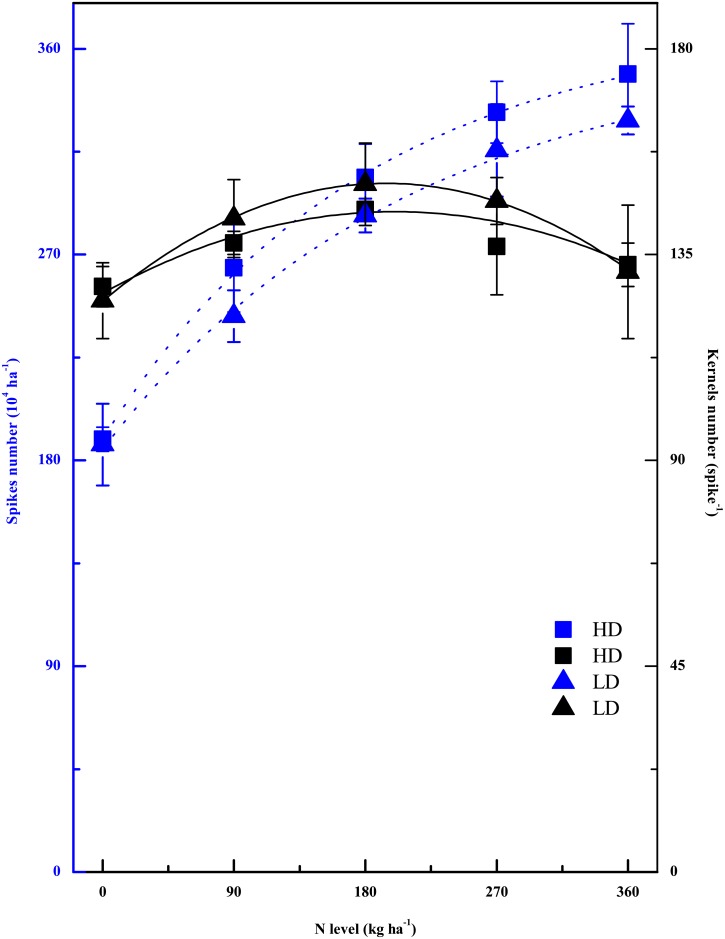
The common coordination of the yield component parameters spike number of per unit area and kernel number of per spike of rice to different N levels and transplant densities.

Rice yield is directly determined by the mutual coordination among yield component parameters. Our results indicated that the highest grain yield at HD was more reliant on the number of spikelets per panicle, while the number of panicles per unit area was more important at LD (Fig 6). However, the numbers of spikelets and panicles were both significantly affected by NL. In a certain range, increasing the NL had positive effects on the number of spikelets per panicle and the number of panicles per unit area, while there were negative effects on the seed setting rate and the 1000-grain weight. Under higher NL (more than 200 kg N ha^-1^), the number of spikelets decreased, while the number of panicles increased continuously with increasing NL. During panicle formation, the biomass accumulation, N uptake and soluble carbohydrate content in the shoot are all essential factors that affect the number of spikelets per panicle, which are all closely related to CPP [32]; The crop growth rate (CGR) during panicle formation is strongly linked to the fill percentage, because lower CGR and N concentration can cause young panicle degeneration during this period [33]. In our experiment, most leaves were still green at the mature stage under higher NLs, indicating that the assimilation of substances exploited by grains as a sink might also be limited by unknown biotic or nutritional factors [34]. However, it remains poorly understood why this phenomenon occurs with nitrogen supply, as the potential candidates were endogenous hormones, *i*.*e*., cytokinins and gibberellins [35–37], which would be altered with changing N levels.

**Fig 7:**
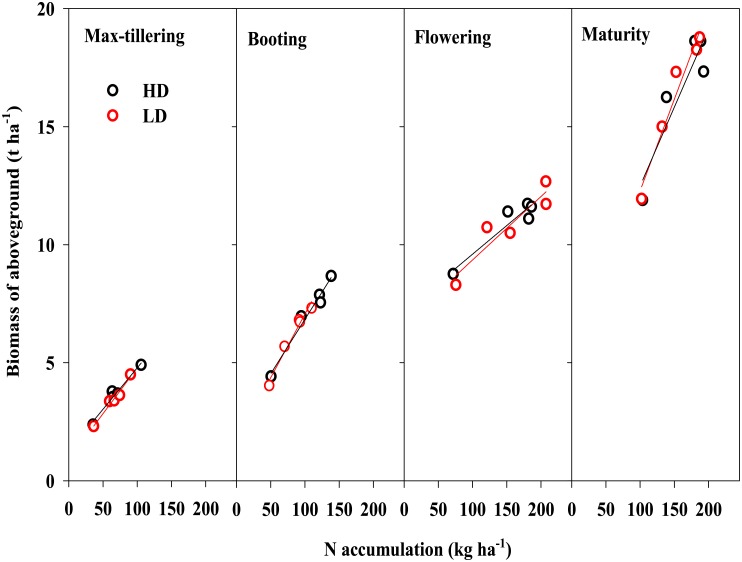
The relationship between N accumulation and biomass of aboveground plant parts at five different growth stages: Max-tillering, booting, flowering, filling and harvest. The data are the mean values.

There was a significant linear correlation between the aboveground biomass and N accumulation during the entire growth period (Fig 7), consistently results have been observed in wheat of different varieties [38]. Both aboveground biomass and N accumulation increased with N supply at each growth stage and achieved their maximum value when NLs were 180 and 270 kg ha^-1^ at HD and LD, respectively (Figs 1 and 3). The responses of rice plant growth to the NL and TD were significantly different during the flowering stage, when accumulation of N and biomass both reached to their maximum value (Fig 7). Studies suggests that soil N significantly affects the growth of crop shoots and slightly affects the roots, especially for root growth at deeper depths [39]. Crop N accumulation is highly related to crop growth rate and biomass accumulation, which rely on the inter-regulation of multiple crop physiological processes including N uptake, C and N assimilation, growth rate, and N and C distribution [20]. Our results demonstrate that to optimize the interaction between the physiological processes and yield, optimal N management and planting density must be considered [12, 14].

## Conclusion

Rice population productivity is regulated by N level and planting density. Our results illustrated that the highest population productivity could be obtained under a higher planting density with a lower N level. The role of N in population productivity could be compensated by planting density, accompanied by more tillers per unit area, higher LAI and more N accumulation of aboveground parts. According to our results, we infer that based on low density, 1 kg N ha^-1^ would be saved by adding approximately 1000 planting hills per hectare with cultivar *Zhendao 11* grown in Rugao Counties, Jiangsu Province, China. Therefore, balance the conflict between higher nutrient input and lower environmental cost for high crop yield and high nitrogen use efficiency, by regulating the planting density is very conducive to the sustainable development of intensive agriculture, which the production concept of the reducing nitrogen rate and increasing plant density could be applied in China and even the worldwide.

## Supporting information

S1 Excel Data for Table 1. (PDF)

S2 Excel Data for Table 2. (PDF)

S3 Excel Data for Fig 1. (PDF)

S4 Excel Data for Fig 2. (PDF)

S5 Excel Data for Fig 3. (PDF)

S6 Excel Data for Fig 4. (PDF)

S7 Excel Data for Fig 5. (PDF)

S8 Excel Data for Fig 6. (PDF)

S9 Excel Data for Fig 7. (PDF)
