# Supplementary material for: Improving rice population productivity by reducing nitrogen rate and increasing plant density
Source: PLoS One. 2017 Aug 2;12(8):e0182310. doi: 10.1371/journal.pone.0182310 (PMC5540556; doi:10.1371/journal.pone.0182310)
Supplement: S1 Excel — (PDF) [file pone.0182310.s001.pdf]

Year	Yield (t/ha)					
2012		1	2	3	AVE	SD
HD	0	6.0	5.9	6.2	6.0	0.2
	90	9.4	8.9	8.8	9.0	0.3
	180	9.0	9.5	9.2	9.2	0.3
	270	9.0	8.8	8.1	8.6	0.5
	360	9.2	9.2	8.3	8.9	0.5
LD	0	6.2	6.2	6.9	6.4	0.4
	90	8.5	9.2	8.4	8.7	0.4
	180	9.2	9.2	9.2	9.2	0.0
	270	9.3	9.4	10.1	9.6	0.4
	360	9.4	8.9	8.6	9.0	0.4
2013	t/ha					
		1	2	3	AVE	SD
HD	0	5.6	5.9	5.7	5.7	0.1
	90	7.3	7.8	7.4	7.5	0.3
	180	8.0	8.2	8.1	8.1	0.1
	270	8.2	8.1	8.1	8.1	0.0
	360	8.5	8.3	8.0	8.3	0.2
LD	0	5.6	5.9	6.1	5.8	0.3
	90	7.6	7.3	7.3	7.4	0.2
	180	7.9	7.6	8.3	7.9	0.4
	270	7.8	8.1	7.6	7.8	0.2
	360	8.4	8.6	8.2	8.4	0.2
2014 Yield	t/ha					
HD		1	2	3	AVE	SD
	0	6.7	5.8	5.5	6.0	0.59
	90	9.2	8.0	8.9	8.7	0.58
	180	10.3	10.3	9.2	9.9	0.65
	270	9.9	9.6	10.2	9.9	0.29
	360	9.4	10.0	9.7	9.7	0.25
LD						
	0	6.5	5.5	5.5	5.8	0.59
	90	8.9	8.5	8.5	8.6	0.22
	180	9.0	9.7	9.4	9.4	0.37
	270	10.0	10.2	9.8	10.0	0.21
	360	10.1	10.0	10.1	10.1	0.05
			Panicles Number (104 ha^{-1})			
2012		1	2	3	AVE	SD
HD	0	187	198	196	194	6

	90	269	295	287	284	13
	180	302	307	352	320	28
	270	323	346	339	336	12
	360	346	378	383	369	20
LD	0	199	209	196	201	7
	90	252	255	246	251	5
	180	287	293	276	285	9
	270	325	339	353	339	14
	360	323	324	341	329	10

2013		1	2	3	AVE	SD
HD	0	201	179	192	191	11
	90	260	276	263	266	9
	180	291	276	305	291	14
	270	311	320	324	318	7
	360	341	354	357	351	9
LD	0	209	185	182	192	15
	90	243	260	243	249	10
	180	276	276	293	282	10
	270	276	308	317	300	21
	360	341	306	357	335	26

2014		1	2	3	AVE	SD
HD	0	188	181	179	183	4
	90	251	238	245	245	6
	180	302	290	314	302	12
	270	343	336	354	344	9
	360	327	320	331	326	5
LD						
	0	165	170	166	167	3
	90	247	213	230	230	17
	180	289	301	299	296	7
	270	328	298	300	309	17
	360	299	344	324	322	22

Spikelets number (panicle^{-1})

2012		1	2	3	AVE	SD
HD	0	125	120	126	124	3
	90	121	131	123	125	5
	180	104	124	108	112	11
	270	140	104	132	125	18
	360	120	102	108	110	9
LD	0	108	104	113	108	5

	90	120	130	133	128	7
	180	128	124	142	131	10
	270	132	112	114	119	11
	360	106	107	105	106	1

2013		1	2	3	AVE	SD
HD	0	137	133	128	132	4.3
	90	145	147	129	140	9.8
	180	139	148	150	146	5.5
	270	141	133	143	139	5.4
	360	137	144	137	139	4.2
LD	0	137	135	138	136	1.6
	90	140	150	152	147	6.5
	180	155	144	142	147	7.0
	270	139	148	142	143	4.5
	360	134	132	132	133	0.9

2014		1	2	3	AVE	SD
HD	0	137	144	137	139	4.3
	90	139	131	135	135	4.3
	180	146	136	151	144	7.6
	270	144	146	148	146	2.4
	360	155	149	145	149	5.1
LD	0	125	135	131	130	5.2
	90	165	165	132	154	18.9
	180	150	166	146	154	10.6
	270	178	175	182	178	3.5
	360	148	149	147	148	1.0

Filled Grains rate（%）

2012		1	2	3	AVE	SD
HD	0	98.7	97.9	98.0	98.2	0.5
	90	98.5	97.7	97.8	98.0	0.5
	180	97.7	97.0	87.0	97.3	0.4
	270	90.7	90.6	91.9	91.0	0.7
	360	93.6	94.0	94.4	94.0	0.4
LD						
	0	98.2	98.7	98.2	98.4	0.3
	90	97.7	97.2	97.6	97.5	0.3
	180	97.8	95.8	96.2	96.6	1.0
	270	93.8	92.1	91.2	92.4	1.3
	360	95.0	94.2	93.0	94.0	1.0

2013

		1	2	3	AVE	SD
HD						
	0	95.8	94.6	95.2	95.2	0.89
	90	97.2	95.8	96.5	96.5	0.97
	180	95.4	94.1	94.7	94.7	0.96
	270	91.5	92.1	91.8	91.8	0.46
	360	91.0	85.5	88.3	88.3	3.94
LD						
	0	96.7	92.2	94.4	94.4	3.13
	90	95.9	91.5	93.7	93.7	3.06
	180	95.8	96.2	96	96.0	0.29
	270	86.7	93.8	90.2	90.2	5.01
	360	90.1	91.9	91	91.0	1.29

2014

		1	2	3	AVE	SD
HD						
	0	97.8	97.8	97.8	97.8	0.0
	90	98.4	96.7	97.6	97.6	1.2
	180	97.8	97.1	98.5	97.8	0.7
	270	97.4	97.4	97.5	97.4	0.1
	360	96.6	94.9	98.1	96.6	1.6
LD						
	0	98.2	98.1	98.2	98.2	0.0
	90	97.8	97.6	98.3	97.9	0.4
	180	97.0	97.0	98.0	97.3	0.6
	270	96.5	95.7	97.2	96.5	1.1
	360	96.2	94.1	98.2	96.2	2.9

1000-Grain Weight(g)

2012		1	2	3	AVE	SD
HD	0	29.8	29.9	29.7	29.8	0.1
	90	27.3	26.9	26.0	26.7	0.7
	180	25.1	27.0	25.4	25.8	1.0
	270	25.5	25.7	25.2	25.5	0.3
	360	25.7	24.9	25.8	25.5	0.5
LD						
	0	30.1	29.3	29.1	29.5	0.6
	90	26.9	26.9	26.9	26.9	0.0
	180	26.2	26.3	26.3	26.3	0.1
	270	24.9	24.9	24.8	24.9	0.1
	360	24.8	24.6	24.7	24.7	0.1

2013		1	2	3	AVE	SD
HD	0	27.1	26.5	26.7	26.8	0.31
	90	26.6	26.6	26.6	26.6	0.00
	180	24.5	25.9	25.5	25.3	0.74
	270	24.7	25.4	25.0	25.0	0.34

	360	23.5	23.1	23.4	23.3	0.22
LD	0	26.7	26.9	26.3	26.6	0.29
	90	26.9	26.7	25.8	26.5	0.61
	180	25.5	25.6	24.9	25.3	0.38
	270	24.2	24.8	25.7	24.9	0.77
	360	23.5	24.8	24.2	24.2	0.62
2014		1	2	3	AVE	SD
HD	0	26.5	26.7	27.0	26.7	0.2
	90	26.5	26.4	26.4	26.5	0.0
	180	26.5	26.4	25.8	26.2	0.4
	270	24.5	22.6	25.2	24.1	1.3
	360	21.9	22.7	22.9	22.5	0.6
LD	0	26.9	27.6	27.8	27.4	0.5
	90	27.2	25.8	26.1	26.4	0.7
	180	28.3	25.8	25.6	26.6	1.5
	270	25.2	24.0	25.8	25.0	0.9
	360	22.6	21.9	23.2	22.6	0.6
