# Supplementary material for: Improving rice population productivity by reducing nitrogen rate and increasing plant density
Source: PLoS One. 2017 Aug 2;12(8):e0182310. doi: 10.1371/journal.pone.0182310 (PMC5540556; doi:10.1371/journal.pone.0182310)
Supplement: S2 Excel — (PDF) [file pone.0182310.s002.pdf]

Leaf N concentration (mg/g)

	HD		1	2	3	AVE	SD
Max-tiller		0	22.1	22.6	24.1	22.9	1.0
		90	26.5	26.5	24.5	25.8	1.1
		180	24.8	24.7	28.2	25.9	2.0
		270	27.4	27.3	27.5	27.4	0.1
		360	28.2	29.8	29.9	29.3	1.0
	LD						
		0	25.1	26.1	22.8	24.6	1.7
		90	26.5	26.5	26.7	26.6	0.1
		180	30.0	28.4	28.6	28.5	0.1
		270	29.6	28.0	30.0	29.2	1.1
		360	25.1	25.5	30.4	27.0	2.9
Booting	HD		1	2	3	AVE	SD
		0	19.5	19.6	20.1	19.7	0.3
		90	24.0	23.4	20.7	22.7	1.7
		180	24.4	23.7	24.3	24.1	0.4
		270	24.1	23.9	27.1	25.1	1.8
		360	26.3	26.9	26.2	26.5	0.4
	LD						
		0	17.6	18.2	20.7	18.8	1.6
		90	22.7	22.9	20.1	22.8	0.2
		180	21.1	21.9	22.8	22.4	0.7
		270	24.7	25.4	25.0	25.0	0.3
		360	27.8	28.1	29.5	28.0	0.2
Flowering	HD		1	2	3	AVE	SD
		0	15.4	17.2	14.9	15.8	1.2
		90	21.6	21.3	22.3	21.7	0.5
		180	24.4	25.5	28.0	25.0	0.7
		270	28.9	28.3	30.7	28.6	0.4
		360	28.2	26.7	31.1	27.4	1.1
	LD						
		0	18.7	19.2	20.0	19.3	0.7
		90	18.7	20.6	20.9	20.1	1.2
		180	28.5	27.2	28.3	28.0	0.7
		270	29.2	28.7	27.2	27.9	1.1
		360	29.6	30.5	33.5	30.0	0.6
Maturity	HD		1	2	3	AVE	SD
		0	5.5	5.0	9.6	5.3	0.3
		90	7.7	8.4	7.2	7.8	0.6

	180	7.6	6.6	7.2	7.1	0.5
	270	7.5	8.7	8.6	8.3	0.7
	360	11.3	8.0	8.4	8.2	0.3
LD						
	0	4.6	6.9	8.7	5.8	1.6
	90	8.1	7.3	7.5	7.6	0.4
	180	9.3	6.9	4.5	8.1	1.7
	270	8.9	7.5	7.7	8.0	0.8
	360	9.0	7.5	9.7	9.4	0.5

Sheath N concentration (mg/g)

HD		1	2	3	AVE	SD
	0	8.6	8.2	10.6	8.4	0.3
	90	10.0	10.8	10.1	10.1	0.1
	180	9.2	9.0	10.4	9.5	0.7
	270	11.0	10.9	11.4	10.9	0.0
	360	11.8	11.7	13.7	11.8	0.1
LD						
	0	8.4	8.8	10.3	8.6	0.3
	90	10.1	10.0	10.0	10.0	0.0
	180	10.5	10.6	11.5	10.9	0.6
	270	10.8	11.3	12.8	11.1	1.0
	360	10.4	10.4	14.8	10.4	0.0

HD		1	2	3	AVE	SD
	0	6.0	5.9	5.5	5.8	0.2
	90	6.2	6.2	6.7	6.4	0.3
	180	8.4	8.3	7.7	8.1	0.4
	270	7.9	8.0	8.6	8.2	0.4
	360	8.7	8.7	8.0	8.5	0.4
LD						
	0	5.5	5.8	6.5	5.9	0.5
	90	7.0	6.6	6.3	6.7	0.3
	180	7.0	7.7	7.8	7.5	0.4
	270	8.3	8.3	7.8	8.1	0.3
	360	9.7	10.4	10.4	10.2	0.4

HD		1	2	3	AVE	SD
	0	4.6	4.9	5.4	5.0	0.4
	90	5.6	6.1	6.9	6.2	0.7
	180	7.4	7.7	9.1	7.6	0.2
	270	8.2	10.9	11.8	11.4	0.6
	360	10.3	11.4	10.4	10.7	0.6
LD						
	0	5.4	5.8	5.3	5.5	0.3

	90	7.0	5.6	6.0	5.8	0.3
	180	8.9	8.4	7.9	8.4	0.5
	270	10.8	10.9	9.3	10.3	0.9
	360	12.2	11.8	11.2	11.5	0.5

HD		1	2	3	AVE	SD
	0	3.0	3.3	3.2	3.2	0.1
	90	3.6	3.2	3.2	3.3	0.2
	180	4.5	4.8	6.0	4.6	0.2
	270	4.2	5.9	6.6	6.2	0.5
	360	7.0	5.9	5.8	6.3	0.7
LD						
	0	2.7	3.6	3.3	3.0	0.4
	90	4.0	2.3	2.9	2.6	0.4
	180	5.9	4.4	3.9	4.1	0.4
	270	4.9	5.0	4.4	4.8	0.4
	360	5.3	6.2	4.9	5.1	0.3

Shoot N concentration (mg/g)

HD		1	2	3	AVE	SD
	0	5.1	4.5	7.0	4.8	0.4
	90	6.4	7.8	7.6	7.3	0.7
	180	7.9	8.6	8.7	8.4	0.4
	270	11.7	10.3	10.4	10.8	0.8
	360	7.5	11.3	12.7	12.0	1.0
LD						
	0	5.3	5.0	6.1	5.5	0.5
	90	8.6	7.2	6.5	7.4	1.0
	180	11.5	8.2	7.4	9.1	2.2
	270	11.4	10.6	9.8	10.6	0.8
	360	15.3	13.5	13.0	13.2	0.4

HD		1	2	3	AVE	SD
	0	5.7	6.2	6.3	6.3	0.1
	90	7.7	6.9	6.8	6.9	0.1
	180	5.0	8.2	5.7	5.4	0.5
	270	7.5	8.1	7.3	7.7	0.4
	360	8.9	8.4	9.1	8.8	0.4
LD						
	0	6.0	6.6	7.1	6.3	0.4
	90	6.5	5.5	7.6	6.0	0.7
	180	5.9	8.8	7.1	6.5	0.8
	270	7.1	6.8	6.8	6.9	0.2
	360	7.3	8.2	8.5	8.0	0.7

Panicles N concentration (mg/g)

HD		1	2	3	AVE	SD
	0	8.3	9.2	9.7	9.1	0.7
	90	10.5	10.8	9.8	10.3	0.5
	180	10.4	11.0	10.8	10.8	0.3
	270	13.0	11.8	10.9	11.9	1.1
	360	12.4	11.4	12.1	12.0	0.5
LD						
	0	9.3	8.6	9.0	9.0	0.4
	90	10.3	9.7	9.9	10.0	0.3
	180	11.0	11.5	10.6	11.0	0.4
	270	13.6	11.7	12.8	12.7	0.9
	360	15.8	13.6	12.5	13.0	0.7

HD		1	2	3	AVE	SD
	0	10.7	10.7	11.7	11.0	0.6
	90	11.7	11.4	11.4	11.5	0.2
	180	12.7	12.8	12.2	12.6	0.3
	270	12.8	14.3	13.8	13.6	0.8
	360	15.2	15.2	13.9	14.8	0.8
LD						
	0	11.2	11.7	10.7	11.2	0.5
	90	12.1	11.3	12.2	11.9	0.5
	180	13.7	12.9	10.9	13.3	1.4
	270	14.5	14.3	13.1	14.0	0.8
	360	14.1	15.3	14.1	14.5	0.7
