# Supplementary material for: Improving rice population productivity by reducing nitrogen rate and increasing plant density
Source: PLoS One. 2017 Aug 2;12(8):e0182310. doi: 10.1371/journal.pone.0182310 (PMC5540556; doi:10.1371/journal.pone.0182310)
Supplement: S3 Excel — (PDF) [file pone.0182310.s003.pdf]

Aboveground biomass (t/ha)

Maximum-tiller stag		1	2	3	AVE	SD
	0	2.55	2.21	3.59	2.38	0.24
	90	3.84	5.90	3.72	3.78	0.09
	180	3.51	3.77	3.33	3.54	0.22
	270	4.93	3.44	3.94	3.69	0.35
	360	5.10	4.18	4.71	4.91	0.28
	0	2.12	2.21	2.59	2.31	0.25
	90	3.12	3.74	3.24	3.37	0.33
	180	3.41	3.42	3.36	3.40	0.03
	270	3.69	2.91	3.56	3.62	0.09
	360	4.92	3.46	4.08	4.50	0.59
	t/ha					
Booting stage		1	2	3	AVE	SD
	0	5.11	4.30	4.53	4.42	0.16
	90	6.48	8.15	7.45	6.96	0.69
	180	7.65	8.10	6.12	7.88	0.32
	270	7.24	7.86	6.61	7.55	0.44
	360	8.92	8.43	4.08	8.68	0.35
	0	3.89	4.16	4.98	4.02	0.19
	90	5.06	6.11	5.89	5.69	0.56
	180	7.03	6.59	5.74	6.81	0.32
	270	8.70	5.84	5.62	6.72	1.72
	360	7.48	7.15	6.49	7.31	0.24
Flowering	t/ha					
		1	2	3	AVE	SD
	0	8.3	7.3	9.2	8.8	0.65
	90	13.5	13.3	13.3	11.4	0.14
	180	11.3	11.5	12.4	11.7	0.59
	270	9.6	16.6	10.7	11.1	0.78
	360	11.2	12.0	14.6	11.6	0.52
	0	7.9	8.5	8.6	8.3	0.38
	90	10.9	11.9	10.6	10.7	0.20
	180	10.2	10.4	10.9	10.5	0.38
	270	12.0	13.3	9.8	12.7	0.92
	360	12.8	11.1	11.3	11.7	0.95
	t/ha					
		1	2	3	AVE	SD
Maturity	0	12.2	10.9	11.6	11.9	0.39

90	16.1	13.6	16.4	16.2	0.28
180	19.6	18.3	18.0	18.6	0.83
270	19.1	17.3	19.5	18.6	1.16
360	17.3	18.1	17.4	17.3	0.11
0	11.6	15.6	12.3	11.9	0.49
90	15.9	14.8	14.2	15.0	0.87
180	17.0	14.6	17.7	17.3	0.48
270	16.9	18.2	19.3	18.8	0.77
360	16.7	16.4	21.7	18.3	2.97
