# Supplementary material for: Improving rice population productivity by reducing nitrogen rate and increasing plant density
Source: PLoS One. 2017 Aug 2;12(8):e0182310. doi: 10.1371/journal.pone.0182310 (PMC5540556; doi:10.1371/journal.pone.0182310)
Supplement: S4 Excel — (PDF) [file pone.0182310.s004.pdf]

Ratio of leaf biomass

Maximum tiller stage

	1	2	3	AVE	SD
0	0.44	0.41	0.42	0.42	0.01
90	0.46	0.46	0.46	0.46	0.00
180	0.48	0.46	0.47	0.47	0.01
270	0.47	0.50	0.54	0.50	0.03
360	0.49	0.49	0.52	0.50	0.01
0	0.43	0.42	0.42	0.42	0.01
90	0.44	0.44	0.44	0.44	0.00
180	0.46	0.46	0.46	0.46	0.00
270	0.49	0.49	0.52	0.50	0.01
360	0.49	0.49	0.50	0.50	0.00

Booting stage

	1	2	3	AVE	SD
0	0.37	0.37	0.36	0.37	0.01
90	0.40	0.39	0.38	0.39	0.01
180	0.45	0.42	0.41	0.43	0.02
270	0.47	0.42	0.44	0.44	0.03
360	0.47	0.45	0.42	0.45	0.02
0	0.38	0.35	0.34	0.36	0.02
90	0.38	0.36	0.37	0.37	0.01
180	0.41	0.41	0.41	0.41	0.00
270	0.44	0.45	0.45	0.45	0.00
360	0.44	0.45	0.45	0.45	0.01

Flowering stage

	1	2	3	AVE	SD
0	0.28	0.24	0.25	0.26	0.02
90	0.33	0.32	0.33	0.33	0.01
180	0.36	0.35	0.34	0.35	0.01
270	0.37	0.36	0.32	0.35	0.03
360	0.40	0.35	0.35	0.37	0.03
0	0.28	0.24	0.22	0.25	0.03
90	0.31	0.31	0.30	0.31	0.01
180	0.35	0.33	0.32	0.33	0.02
270	0.38	0.35	0.34	0.36	0.02
360	0.38	0.37	0.36	0.37	0.01

Maximun	AVE	SD	AVE	SD	
0	0.42	0.01	0.42	0.01	0.42-0.50
90	0.46	0.01	0.44	0.01	
180	0.47	0.01	0.46	0.01	
270	0.50	0.03	0.50	0.01	

360	0.50	0.01	0.50	0.00	
Booting stage					
0	0.37	0.01	0.36	0.02	0.36-0.45
90	0.39	0.01	0.37	0.01	
180	0.43	0.02	0.41	0.00	
270	0.44	0.03	0.45	0.00	
360	0.45	0.02	0.45	0.01	
Flowering stage					
0	0.26	0.02	0.25	0.03	0.25-0.37
90	0.33	0.01	0.31	0.01	
180	0.35	0.01	0.33	0.02	
270	0.35	0.03	0.36	0.02	
360	0.37	0.03	0.37	0.01	

Leaf area index Maximum tiller stage

H	1	2	3	AVE	SD
0	2.60	2.60	2.36	2.52	0.14
90	3.23	3.17	3.28	3.23	0.06
180	3.83	3.71	3.82	3.79	0.07
270	3.73	4.19	4.62	4.18	0.44
360	4.93	5.19	4.83	4.98	0.19
0	2.08	2.15	2.57	2.27	0.27
90	3.14	2.86	2.81	2.93	0.18
180	3.49	3.50	3.58	3.52	0.05
270	4.07	4.04	4.19	4.10	0.08
360	4.89	5.27	4.61	4.93	0.33

Booting stage

H	1	2	3	AVE	SD
0	3.25	3.21	3.58	3.35	0.20
90	4.90	4.63	4.89	4.81	0.15
180	6.13	6.23	6.21	6.19	0.05
270	6.47	7.05	7.49	7.00	0.51
360	8.18	7.66	7.88	7.77	0.15
0	2.53	3.05	3.33	2.97	0.41
90	3.57	3.99	4.24	3.93	0.34
180	5.74	5.47	5.5	5.57	0.15
270	6.60	7.05	6.69	6.78	0.24
360	7.71	7.62	7.55	7.63	0.08

Flowering stage

H	1	2	3	4	AVE	SD
0	2.98	3.38	3.57	4.29	3.55	0.55
90	3.61	3.96	4.86	4.73	4.29	0.61
180	6.07	6.54	5.76	5.80	6.04	0.36
270	6.79	7.04	7.25	7.02	7.02	0.19

360	7.68	6.22	5.97	6.62	6.62	0.76
0	2.73	3.81	3.03	3.75	3.33	0.53
90	3.69	3.73	4.82	4.08	4.08	0.52
180	5.51	6.02	6.06	5.71	5.83	0.26
270	8.40	8.59	6.77	6.00	7.44	1.26
360	7.54	8.01	7.49	8.55	7.90	0.49

Maximum	HD	LD	HD	LD
	AVE	AVE	SD	SD
0	2.52	0.14	2.27	0.27
90	3.23	0.06	2.93	0.18
180	3.79	0.07	3.52	0.05
270	4.18	0.44	4.10	0.08
360	4.98	0.19	4.93	0.33
Booting stage				
0	3.35	0.20	2.97	0.41
90	4.81	0.15	3.93	0.34
180	6.19	0.05	5.57	0.15
270	7.00	0.51	6.78	0.24
360	7.77	0.15	7.63	0.08
Flowering stage				
0	3.55	0.55	3.33	0.53
90	4.29	0.61	4.08	0.52
180	6.04	0.36	5.83	0.26
270	7.02	0.19	7.44	1.26
360	6.62	0.76	7.90	0.49

Pn*LAI $molCO_2ha^{-1}S^{-1}$

Maximum tiller stage

	1	2	3	AVE	SD
0	0.52	0.56	0.56	0.54	0.03
90	0.81	0.85	0.71	0.79	0.08
180	0.91	1.01	0.86	0.93	0.08
270	1.09	0.93	0.86	0.96	0.12
360	0.95	1.09	1.15	1.06	0.10
0	0.43	0.51	0.45	0.46	0.04
90	0.62	0.72	0.75	0.70	0.07
180	0.82	0.80	0.69	0.77	0.07
270	0.91	0.92	0.96	0.93	0.03
360	1.04	1.11	1.04	1.07	0.04

Booting st	1	2	3	AVE	SD

0	0.66	0.70	0.72	0.69	0.03
90	1.08	1.14	1.15	1.12	0.04
180	1.50	1.53	1.48	1.50	0.02
270	1.60	1.64	1.72	1.62	0.03
360	1.93	1.86	1.90	1.88	0.02
0	0.61	0.69	0.72	0.67	0.05
90	0.82	0.96	0.99	0.92	0.09
180	1.32	1.44	1.34	1.37	0.06
270	1.61	1.38	1.49	1.55	0.09
360	1.66	1.76	1.72	1.74	0.03

Flowering Pn*LAI $molCO_2ha^{-1}S^{-1}$

	1	2	3	4	AVE	SD
0	0.41	0.50	0.51	0.61	0.54	0.06
90	0.64	0.70	0.87	0.84	0.81	0.09
180	1.07	1.12	0.94	0.99	1.09	0.04
270	1.11	1.11	1.14	1.12	1.12	0.01
360	1.32	1.04	0.93	1.09	1.15	0.15
0	0.34	0.50	0.43	0.50	0.48	0.04
90	0.62	0.58	0.74	0.65	0.65	0.06
180	1.00	1.08	0.98	1.00	0.99	0.01
270	1.47	1.48	1.23	1.06	1.39	0.14
360	1.36	1.32	1.29	1.48	1.33	0.04

	HD	LD	HD	LD
Maximun	AVE	AVE	SD	SD
0	0.54	0.03	0.46	0.04
90	0.79	0.08	0.70	0.07
180	0.93	0.08	0.77	0.07
270	0.96	0.12	0.93	0.03
360	1.06	0.10	1.07	0.04
Booting stage				
0	0.69	0.03	0.67	0.05
90	1.12	0.04	0.92	0.09
180	1.50	0.02	1.37	0.06
270	1.62	0.03	1.55	0.09
360	1.88	0.02	1.74	0.03
Flowering stage				
0	0.54	0.06	0.48	0.04
90	0.81	0.09	0.65	0.06
180	1.09	0.04	0.99	0.01
270	1.12	0.01	1.39	0.14
360	1.15	0.15	1.33	0.04
