# Supplementary material for: Improving rice population productivity by reducing nitrogen rate and increasing plant density
Source: PLoS One. 2017 Aug 2;12(8):e0182310. doi: 10.1371/journal.pone.0182310 (PMC5540556; doi:10.1371/journal.pone.0182310)
Supplement: S5 Excel — (PDF) [file pone.0182310.s005.pdf]

Total N accumulation (kg/ha)

Maximum-tillier

HD	1	2	3	4	AVE	SD
0	37	32	36	37	35	2.4
90	67	63	62	62	63	2.3
180	65	64	62	65	64	1.6
270	72	64	74	76	71	4.9
360	105	106	102	110	106	3.5
LD						
0	33	36	41	35	36	3.2
90	54	65	59	60	59	4.4
180	66	66	65	67	66	0.6
270	75	74	73	75	74	0.8
360	87	90	92	91	90	2.1

Booting

HD	1	2	3	4	AVE	SD
0	55	48	51	48	50	3.4
90	85	107	93	93	95	9.1
180	119	124	122	120	121	2.3
270	112	122	132	125	123	8.4
360	151	145	117	141	138	14.7
LD						
0	39	43	59	50	48	8.5
90	65	80	71	65	70	7.0
180	91	95	83	97	91	6.3
270	100	91	84	95	93	6.7
360	113	108	111	107	110	2.6

Flowering

HD	1	2	3	4	AVE	SD
0	65	69	82	71	72	7.2
90	143	155	155	153	152	5.7
180	173	185	189	174	180	8.0
270	165	175	185	203	182	16.3
360	186	201	177	181	186	10.4
LD						
0	72	78	80	73	76	4.0
90	119	132	115	118	121	7.8
180	158	150	160	152	155	4.6
270	212	229	200	188	207	17.2
360	210	201	211	208	207	4.5

Maturity

HD	1	2	3	AVE	SD

0	96	101	114	104	9.4
90	147	123	146	139	13.2
180	188	178	174	180	7.0
270	181	190	195	189	7.4
360	204	184	191	193	10.0
LD					
0	95	102	109	102	6.6
90	136	118	142	132	12.3
180	164	149	145	153	10.0
270	180	187	194	187	7.4
360	180	192	175	183	8.6
