# Supplementary material for: Improving rice population productivity by reducing nitrogen rate and increasing plant density
Source: PLoS One. 2017 Aug 2;12(8):e0182310. doi: 10.1371/journal.pone.0182310 (PMC5540556; doi:10.1371/journal.pone.0182310)
Supplement: S6 Excel — (PDF) [file pone.0182310.s006.pdf]

		YC_N (%)				
2012		1	2	3	AVE	SD
HD	0					
	90	36.3	34.4	29.2	33.3	3.7
	180	33.3	38.5	32.1	34.6	3.4
	270	33.3	33.2	23.3	29.9	5.7
	360	34.4	36.0	25.4	32.0	5.7
LD	0					
	90	27.0	32.4	18.7	29.7	3.8
	180	32.4	32.0	25.5	30.0	3.9
	270	33.5	33.5	32.2	33.1	0.8
	360	34.2	29.9	20.5	32.0	3.1

2013		YC_N (%)				
		1	2	3	AVE	SD
HD	0					
	90	26.3	13.8	22.2	24.2	2.9
	180	30.9	28.9	29.2	29.9	1.5
	270	31.8	27.9	29.2	29.6	2.0
	360	33.9	29.6	28.8	31.3	2.8
LD	0					
	90	26.6	20.4	16.3	21.5	5.2
	180	29.1	23.0	26.7	26.3	3.1
	270	28.1	25.0	22.0	25.0	3.0
	360	34.0	31.8	26.1	29.0	4.1

2014		YC_N (%)				
HD		1	2	3	AVE	SD
	0					
	90	27.1	27.3	37.8	30.7	6.1
	180	34.9	43.1	39.5	39.2	4.1
	270	32.6	39.0	45.5	39.0	6.5
	360	29.3	41.2	43.0	42.1	1.2
LD						
	0					
	90	26.6	35.3	35.4	32.5	5.1
	180	27.3	43.0	42.0	37.4	8.8
	270	34.6	45.8	44.2	45.0	1.2
	360	35.7	44.9	45.9	45.4	0.7

		YC_N (%)				
2012		1	2	3	AVE	SD

HD	0					
	90	38.0	34.2	28.4	33.5	4.8
	180	16.6	20.4	16.3	17.8	2.3
	270	11.1	10.8	7.0	10.9	0.2
	360	8.8	9.2	5.9	9.0	0.3
LD	0					
	90	25.6	33.3	17.6	29.4	5.5
	180	16.6	16.3	13.1	15.3	2.0
	270	11.6	11.7	12.1	11.8	0.3
	360	9.0	7.4	4.9	8.2	1.1
2013			YC_N (%)			
		1	2	3	AVE	SD
HD	0					
	90	22.1	10.4	18.2	20.2	2.8
	180	13.9	13.2	13.1	13.4	0.4
	270	9.7	8.4	8.8	8.9	0.7
	360	8.0	6.8	6.4	7.2	1.1
LD	0					
	90	22.5	16.6	13.2	22.5	4.7
	180	12.7	9.7	12.3	11.6	1.6
	270	8.1	7.2	6.3	7.2	0.9
	360	8.0	7.6	6.0	6.8	1.1
2014			YC_N (%)			
HD		1	2	3	AVE	SD
	0					
	90	27.7	24.4	37.3	26.0	2.3
	180	19.9	24.6	20.1	21.5	2.7
	270	12.0	13.8	17.1	12.9	1.3
	360	7.7	11.4	11.6	10.2	2.2
LD						
	0					
	90	26.3	33.5	33.3	31.0	4.1
	180	13.6	23.2	22.0	19.6	5.2
	270	12.8	17.3	16.0	15.4	2.3
	360	10.0	12.5	12.9	11.8	1.5
