# Supplementary material for: Improving rice population productivity by reducing nitrogen rate and increasing plant density
Source: PLoS One. 2017 Aug 2;12(8):e0182310. doi: 10.1371/journal.pone.0182310 (PMC5540556; doi:10.1371/journal.pone.0182310)
Supplement: S7 Excel — (PDF) [file pone.0182310.s007.pdf]

| Year | Yield | t/ha | | | | |
|---|---|---|---|---|---|---|
| 2012 | | 1 | 2 | 3 | AVE | SD |
| HD | 0 | 6.0 | 5.9 | 6.2 | 6.0 | 0.2 |
| | 90 | 9.4 | 8.9 | 8.8 | 9.0 | 0.3 |
| | 180 | 9.0 | 9.5 | 9.2 | 9.2 | 0.3 |
| | 270 | 9.0 | 8.8 | 8.1 | 8.6 | 0.5 |
| | 360 | 9.2 | 9.2 | 8.3 | 8.9 | 0.5 |
| | | | | | | |
| LD | 0 | 6.2 | 6.2 | 6.9 | 6.4 | 0.4 |
| | 90 | 8.5 | 9.2 | 8.4 | 8.7 | 0.4 |
| | 180 | 9.2 | 9.2 | 9.2 | 9.2 | 0.0 |
| | 270 | 9.3 | 9.4 | 10.1 | 9.6 | 0.4 |
| | 360 | 9.4 | 8.9 | 8.6 | 9.0 | 0.4 |

| 2013 | | t/ha | | | | |
|---|---|---|---|---|---|---|
| | | 1 | 2 | 3 | AVE | SD |
| HD | 0 | 5.6 | 5.9 | 5.7 | 5.7 | 0.1 |
| | 90 | 7.3 | 7.8 | 7.4 | 7.5 | 0.3 |
| | 180 | 8.0 | 8.2 | 8.1 | 8.1 | 0.1 |
| | 270 | 8.2 | 8.1 | 8.1 | 8.1 | 0.0 |
| | 360 | 8.5 | 8.3 | 8.0 | 8.3 | 0.2 |
| | | | | | | |
| LD | 0 | 5.6 | 5.9 | 6.1 | 5.8 | 0.3 |
| | 90 | 7.6 | 7.3 | 7.3 | 7.4 | 0.2 |
| | 180 | 7.9 | 7.6 | 8.3 | 7.9 | 0.4 |
| | 270 | 7.8 | 8.1 | 7.6 | 7.8 | 0.2 |
| | 360 | 8.4 | 8.6 | 8.2 | 8.4 | 0.2 |

| 2014 | Yield | t/ha | | | | |
|---|---|---|---|---|---|---|
| HD | | 1 | 2 | 3 | AVE | SD |
| | 0 | 6.7 | 5.8 | 5.5 | 6.0 | 0.59 |
| | 90 | 9.2 | 8.0 | 8.9 | 8.7 | 0.58 |
| | 180 | 10.3 | 10.3 | 9.2 | 9.9 | 0.65 |
| | 270 | 9.9 | 9.6 | 10.2 | 9.9 | 0.29 |
| | 360 | 9.4 | 10.0 | 9.7 | 9.7 | 0.25 |
| LD | | | | | | |
| | 0 | 6.5 | 5.5 | 5.5 | 5.8 | 0.59 |
| | 90 | 8.9 | 8.5 | 8.5 | 8.6 | 0.22 |
| | 180 | 9.0 | 9.7 | 9.4 | 9.4 | 0.37 |
| | 270 | 10.0 | 10.2 | 9.8 | 10.0 | 0.21 |
| | 360 | 10.1 | 10.0 | 10.1 | 10.1 | 0.05 |

| | | | | | | |
|---|---|---|---|---|---|---|
| 2012 | HD | | | | Obtain auxiliary point by SAS software | |
| | | 0 | 6000 | 6.0 | 0 | 6 |
| | | 90 | 8900 | 8.9 | 89.53 | 8.9 |
| | | 180 | 9200 | 9.2 | | |
| | | 270 | 8600 | 8.6 | 89.53 | 8.9 |
| | | 360 | 8900 | 8.9 | 360 | 8.9 |
| | LD | | | | | |
| | | 0 | 6200 | 6.2 | 0 | 6.2 |
| | | 90 | 8500 | 8.5 | 120 | 9.27 |
| | | 180 | 9200 | 9.2 | | |
| | | 270 | 9600 | 9.6 | 120 | 9.27 |
| | | 360 | 9000 | 9.0 | 360 | 9.27 |
| 2013 | HD | | | | | |
| | | 0 | 5700 | 5.7 | 0 | 5.7 |
| | | 90 | 7500 | 7.5 | 123 | 8.166 |
| | | 180 | 8100 | 8.1 | | |
| | | 270 | 8100 | 8.1 | 123 | 8.166 |
| | | 360 | 8300 | 8.3 | 360 | 8.166 |
| | LD | | | | | |
| | | 0 | 5700 | 5.8 | 0 | 5.8 |
| | | 90 | 7400 | 7.4 | 192 | 8.25 |
| | | 180 | 7900 | 7.9 | | |
| | | 270 | 8100 | 7.8 | 192 | 8.25 |
| | | 360 | 8400 | 8.4 | 360 | 8.25 |
| 2014 | HD | | | | | |
| | | 0 | 6000 | 6.0 | 0 | 6 |
| | | 90 | 8700 | 8.7 | 127.778 | 9.8 |
| | | 180 | 9900 | 9.9 | | |
| | | 270 | 9900 | 9.9 | 127.778 | 9.8 |
| | | 360 | 9700 | 9.7 | 360 | 9.8 |
| | LD | | | | | |
| | | 0 | 5800 | 5.8 | 0 | 6.1 |
| | | 90 | 8600 | 8.6 | 195.8 | 10.05 |
| | | 180 | 9400 | 9.4 | | |
| | | 270 | 10000 | 10.0 | 195.8 | 10.05 |
| | | 360 | 10100 | 10.1 | 360 | 10.05 |
