# Supplementary material for: Improving rice population productivity by reducing nitrogen rate and increasing plant density
Source: PLoS One. 2017 Aug 2;12(8):e0182310. doi: 10.1371/journal.pone.0182310 (PMC5540556; doi:10.1371/journal.pone.0182310)
Supplement: S8 Excel — (PDF) [file pone.0182310.s008.pdf]

2012	Panicles Number (10^4 ha^{-1})				
HD	1	2	3	AVE	SD
0	187	198	196	194	6
90	269	295	287	284	13
180	302	307	352	320	28
270	323	346	339	336	12
360	346	378	383	369	20
LD					
0	199	209	196	201	7
90	252	255	246	251	5
180	287	293	276	285	9
270	325	339	353	339	14
360	323	324	341	329	10

2013					
HD	1	2	3	AVE	SD
0	201	179	192	191	11
90	260	276	263	266	9
180	291	276	305	291	14
270	311	320	324	318	7
360	341	354	357	351	9
LD					
0	209	185	182	192	15
90	243	260	243	249	10
180	276	276	293	282	10
270	276	308	317	300	21
360	341	306	357	335	26

2014					
HD	1	2	3	AVE	SD
0	188	181	179	183	4
90	251	238	245	245	6
180	302	290	314	302	12
270	343	336	354	344	9
360	327	320	331	326	5
0	165	170	166	167	3
90	247	213	230	230	17
180	289	301	299	296	7
270	328	298	300	309	17
360	299	344	324	322	22

Panicles Number (10^4 ha^{-1})

Average value for three years

HD	1	2	3	AVE	SD
0	192	186	189	189	3.0
90	260	270	265	265	5.0
180	298	291	324	304	17.2
270	326	334	339	333	6.9
360	338	351	357	349	9.9
LD					
0	191	188	181	187	5.0
90	248	242	240	243	4.0
180	284	290	289	288	3.3
270	310	315	323	316	6.8
360	321	325	341	329	10.4

Spikelets number (panicle^{-1})

HD	1	2	3	AVE	SD
0	125	120	126	124	3
90	121	131	123	125	5
180	104	124	108	112	11
270	140	104	132	125	18
360	120	102	108	110	9
LD					
0	108	104	113	108	5
90	120	130	133	128	7
180	128	124	142	131	10
270	132	112	114	119	11
360	106	107	105	106	1

HD					
0	137	133	128	132	4.3
90	145	147	129	140	9.8
180	139	148	150	146	5.5
270	141	133	143	139	5.4
360	137	144	137	139	4.2
LD					
0	137	135	138	136	1.6
90	140	150	152	147	6.5
180	155	144	142	147	7.0
270	139	148	142	143	4.5
360	134	132	132	133	0.9

穗粒数

HD	1	2	3	AVE	SD
0	137	144	137	139	4.3
90	139	131	135	135	4.3
180	146	136	151	144	7.6

	270	144	146	148	146	2.4
	360	155	149	145	149	5.1
LD						
	0	125	135	131	130	5.2
	90	165	165	132	154	18.9
	180	150	166	146	154	10.6
	270	178	175	182	178	3.5
	360	148	149	147	148	1.0

Spikelets number (panicle^{-1})

Average value for three years

HD		1	2	3	AVE	SD
	0	133	132	130	132	1.3
	90	135	136	129	133	3.9
	180	130	136	136	134	3.8
	270	142	128	141	137	7.8
	360	137	132	130	133	3.9
LD						
	0	123	124	127	125	2.3
	90	142	148	139	143	4.6
	180	144	144	143	144	0.5
	270	149	145	146	147	2.4
	360	129	130	128	129	0.7
