# Supplementary material for: Improving rice population productivity by reducing nitrogen rate and increasing plant density
Source: PLoS One. 2017 Aug 2;12(8):e0182310. doi: 10.1371/journal.pone.0182310 (PMC5540556; doi:10.1371/journal.pone.0182310)
Supplement: S9 Excel — (PDF) [file pone.0182310.s009.pdf]

Aboveground biomass (t/ha)

	Max-tillering stage				
HD	1	2	3	AVE	SD
0	2.55	2.21	3.59	2.38	0.24
90	3.84	5.90	3.72	3.78	0.09
180	3.51	3.77	3.33	3.54	0.22
270	4.93	3.44	3.94	3.69	0.35
360	5.10	4.18	4.71	4.91	0.28
LD					
0	2.12	2.21	2.59	2.31	0.25
90	3.12	3.74	3.24	3.37	0.33
180	3.41	3.42	3.36	3.40	0.03
270	3.69	2.91	3.56	3.62	0.09
360	4.92	3.46	4.08	4.50	0.59
	Booting stage				
	1	2	3	AVE	SD
0	5.11	4.30	4.53	4.42	0.16
90	6.48	8.15	7.45	6.96	0.69
180	7.65	8.10	6.12	7.88	0.32
270	7.24	7.86	6.61	7.55	0.44
360	8.92	8.43	4.08	8.68	0.35
0	3.89	4.16	4.98	4.02	0.19
90	5.06	6.11	5.89	5.69	0.56
180	7.03	6.59	5.74	6.81	0.32
270	8.70	5.84	5.62	6.72	1.72
360	7.48	7.15	6.49	7.31	0.24
	Flowering stage				
	1	2	3	AVE	SD
0	8.3	7.3	9.2	8.8	0.65
90	13.5	13.3	13.3	13.4	0.14
180	11.3	11.5	12.4	11.7	0.59
270	9.6	16.6	10.7	10.1	0.78
360	11.2	12.0	14.6	11.6	0.52
0	7.9	8.5	8.6	8.3	0.38
90	10.9	11.9	10.6	10.7	0.20
180	10.2	10.4	10.9	10.5	0.38
270	12.0	13.3	9.8	12.7	0.92
360	12.8	11.1	11.3	11.7	0.95

Maturity

	1	2	3	AVE	SD
0	12.2	10.9	11.6	11.9	0.39
90	16.1	13.6	16.4	16.2	0.28
180	19.6	18.3	18.0	18.6	0.83
270	19.1	17.3	19.5	18.6	1.16
360	17.3	18.1	17.4	17.3	0.11
0	11.6	15.6	12.3	11.9	0.49
90	15.9	14.8	14.2	15.0	0.87
180	17.0	14.6	17.7	17.3	0.48
270	16.9	18.2	19.3	18.8	0.77
360	16.7	16.4	21.7	18.3	2.97

Total N accumulation (kg/ha)

Max-tillering stage

HD	1	2	3	4	AVE	SD
0	37	32	36	37	35	2.4
90	67	63	62	62	63	2.3
180	65	64	62	65	64	1.6
270	72	64	74	76	71	4.9
360	105	106	102	110	106	3.5
LD						
0	33	36	41	35	36	3.2
90	54	65	59	60	59	4.4
180	66	66	65	67	66	0.6
270	75	74	73	75	74	0.8
360	87	90	92	91	90	2.1

Booting stage

HD	1	2	3	4	AVE	SD
0	55	48	51	48	50	3.4
90	85	107	93	93	95	9.1
180	119	124	122	120	121	2.3
270	112	122	132	125	123	8.4
360	151	145	117	141	138	14.7
LD						
0	39	43	59	50	48	8.5
90	65	80	71	65	70	7.0
180	91	95	83	97	91	6.3
270	100	91	84	95	93	6.7
360	113	108	111	107	110	2.6

Flowering stage

HD	1	2	3	4	AVE	SD

0	65	69	82	71	72	7.2
90	143	155	155	153	152	5.7
180	173	185	189	174	180	8.0
270	165	175	185	203	182	16.3
360	186	201	177	181	186	10.4
LD						
0	72	78	80	73	76	4.0
90	119	132	115	118	121	7.8
180	158	150	160	152	155	4.6
270	212	229	200	188	207	17.2
360	210	201	211	208	207	4.5

	Maturity				
HD	1	2	3	AVE	SD
0	96	101	114	104	9.4
90	147	123	146	139	13.2
180	188	178	174	180	7.0
270	181	190	195	189	7.4
360	204	184	191	193	10.0
LD					
0	95	102	109	102	6.6
90	136	118	142	132	12.3
180	164	149	145	153	10.0
270	180	187	194	187	7.4
360	180	192	175	183	8.6
